# Working Memory Training and Speech in Noise Comprehension in Older Adults

**DOI:** 10.3389/fnagi.2016.00049

**Published:** 2016-03-22

**Authors:** Rachel V. Wayne, Cheryl Hamilton, Julia Jones Huyck, Ingrid S. Johnsrude

**Affiliations:** ^1^Department of Psychology, Queen's UniversityKingston, ON, Canada; ^2^Speech Pathology and Audiology, Kent State UniversityKent, OH, USA; ^3^Department of Psychology, School of Communication Sciences and Disorders, The Brain and Mind Institute, University of Western OntarioLondon, ON, Canada

**Keywords:** cognitive training, Cogmed, working memory training, speech-in-noise, speech perception, reading span

## Abstract

Understanding speech in the presence of background sound can be challenging for older adults. Speech comprehension in noise appears to depend on working memory and executive-control processes (e.g., Heald and Nusbaum, 2014), and their augmentation through training may have rehabilitative potential for age-related hearing loss. We examined the efficacy of adaptive working-memory training (Cogmed; Klingberg et al., 2002) in 24 older adults, assessing generalization to other working-memory tasks (near-transfer) and to other cognitive domains (far-transfer) using a cognitive test battery, including the Reading Span test, sensitive to working memory (e.g., Daneman and Carpenter, 1980). We also assessed far transfer to speech-in-noise performance, including a closed-set sentence task (Kidd et al., 2008). To examine the effect of cognitive training on benefit obtained from semantic context, we also assessed transfer to open-set sentences; half were semantically coherent (high-context) and half were semantically anomalous (low-context). Subjects completed 25 sessions (0.5–1 h each; 5 sessions/week) of both adaptive working memory training and placebo training over 10 weeks in a crossover design. Subjects' scores on the adaptive working-memory training tasks improved as a result of training. However, training did not transfer to other working memory tasks, nor to tasks recruiting other cognitive domains. We did not observe any training-related improvement in speech-in-noise performance. Measures of working memory correlated with the intelligibility of low-context, but not high-context, sentences, suggesting that sentence context may reduce the load on working memory. The Reading Span test significantly correlated only with a test of visual episodic memory, suggesting that the Reading Span test is not a pure-test of working memory, as is commonly assumed.

## Introduction

Perception and comprehension of speech heard in background noise becomes more difficult with age (Plomp and Mimpen, 1979; Van Rooij and Plomp, 1990; Sommers, 1997; Schneider et al., 2002; Pichora-Fuller and Souza, 2003). These difficulties are not fully explained by pure-tone audiometric thresholds (hearing sensitivity), and may be due in part to declining frequency selectivity and temporal coding with age (Kujawa and Liberman, 2009; Gordon-Salant et al., 2010; Humes and Dubno, 2010; Plack et al., 2014). Moreover, amplification devices (i.e., hearing aids), the most widely prescribed treatment for hearing difficulties, improve hearing sensitivity but not frequency selectivity or temporal coding, which are important for segregating speech from background sound (e.g., Perez et al., 2014), and many individuals who have been prescribed hearing aids do not wear them (see McCormack and Fortnum, 2013 for a review). As communication difficulties are linked to depression, isolation, and decreased quality of life (Mulrow et al., 1990; Carabellese et al., 1993; Cacciatore et al., 1999), rehabilitative strategies, used either in isolation or in combination with amplification, are urgently needed. Here, we focus on the utility of cognitive strategies for hearing loss rehabilitation.

Hearing loss leads to degradation of the incoming acoustic signal, and the resulting perceptual ambiguity places increased demands on executive processes that mediate knowledge-guided perceptual processes, such as use of context in order to select the contextually appropriate meaning from among competing alternatives (Rodd et al., 2005, 2012; Zekveld et al., 2012). This requires listeners to rely more heavily on top-down information, recruiting previous experience, and linguistic knowledge to help evaluate perceptual hypotheses about the incoming signal (Kane and Engle, 2000).

The ability to use contextual information effectively to enhance intelligibility varies widely among individuals (Davis et al., 2011; Janse and Jesse, 2014). This variability may be attributable, in part, to individual differences in more domain-general cognitive abilities such as processing speed, and executive functions such as working memory and inhibition (for reviews, see Wingfield and Tun, 2007; Arlinger et al., 2009; Schneider et al., 2010; Heald and Nusbaum, 2014). Executive functions allow listeners to direct attention to a particular speaker, integrate the acoustic signal with previous knowledge, and inhibit irrelevant information (e.g., Tun et al., 2002; Woods et al., 2013; Tamati et al., 2013). In addition, knowledge about linguistic structure and sentence parsing may facilitate the use of contextual information to support speech understanding (Rodd et al., 2005; Aydelott et al., 2011; Billig et al., 2013).

Slower processing speed is linked to difficulty understanding speech in noise (e.g., Tun and Wingfield, 1999; Pronk et al., 2013) and may be particularly important for understanding speech spoken at fast rates (e.g., Wingfield et al., 1999; Gordon-Salant and Fitzgibbons, 2001). Both processing speed (Salthouse, 1996) and executive functions (Craik and Salthouse, 2007) decline with age and such declines appear to contribute to listening difficulties in older adults (Humes and Dubno, 2010).

A large body of research has linked working memory measures to speech comprehension in poor listening conditions (due to noise or pathology; e.g., Humes et al., 2006; George et al., 2007; Wingfield and Tun, 2007; Akeroyd, 2008; Rudner et al., 2011; Sorqvist and Rönnberg, 2012; Szenkovits et al., 2012; Zekveld et al., 2012; Tamati et al., 2013; Heald and Nusbaum, 2014; Rudner and Lunner, 2014). The evidence to date supporting the role of working memory in speech perception has been largely correlational. However, working memory may contribute to the ability to use sentence context to guide and constrain interpretation to compensate for increased processing demands when the signal is degraded and interpretation is therefore ambiguous (Rodd et al., 2005, 2012; Zekveld et al., 2012). Thus, individuals with greater working memory capacity may be better able to compensate for degraded listening conditions.

Research linking working memory capacity to speech comprehension has largely relied on the Reading Span test (Daneman and Carpenter, 1980) as a measure of working memory (Tun et al., 1991; Akeroyd, 2008; Zekveld et al., 2012; Besser et al., 2013; Zekveld et al., 2013; Davies-Venn and Souza, 2014, but see Humes and Coughlin, 2009; Schoof and Rosen, 2014). The Rönnberg et al. (1989) version (adapted from Daneman and Carpenter, 1980; Baddeley et al., 1985, but see also Conway et al., 2002; Towse et al., 2008 for alternate versions) is most widely used. These five versions differ in a number of ways (see Table 1). In the canonical Daneman and Carpenter (1980) version (and similarly in the Rönnberg et al., 1989 version), participants read or hear a set of sentences, and are required to make a yes/no semantic judgment after each sentence to prevent rehearsal of items. After each set participants are asked to recall the last word from each sentence in the set in serial order. (See also Lyxell and Rönnberg, 1993, in which subjects are cued whether to recall the first or last word in a set). As the test progresses, the set size increases. Although the Reading Span test is commonly referred to as a test of working memory, it also draws on other cognitive abilities, including processing speed, executive functioning (e.g., selective attention, inhibition, task switching), and reading skill: it is not a “pure test” of working memory. Thus, it is possible that a correlation between working memory and speech in noise (Humes et al., 2006; George et al., 2007; Wingfield and Tun, 2007; Akeroyd, 2008; Rudner et al., 2011; Sorqvist and Rönnberg, 2012; Szenkovits et al., 2012; Zekveld et al., 2012; Tamati et al., 2013; Heald and Nusbaum, 2014; Rudner and Lunner, 2014) may, in fact, be due to other cognitive processes.

**Table 1:** **Description of different versions of the Reading Span test**.

	**Mode of Delivery**	**Recall Item**	**Judgment Task**	**Discontinue Criteria**	**Dependent Variable**
Daneman and Carpenter, 1980	Oral or read from card	Final word in series	Semantic (yes/no)	Fail all three items in set	Span at which items are correctly reported for 2/3 sentences in a set
Baddeley et al., 1985	Oral	Subject or object of sentence (as cued)	Factual (true/false)	N/A	# of items recalled in correct serial order
Rönnberg et al., 1989	Read from computer	Final word in series	Semantic (yes/no)	N/A	# Correct items recalled/maximum score
Conway et al., 2002	Read from computer	Unrelated word presented at end of each sentence	None; must score better than 50% on comprehension post-test	N/A	# of items recalled in correct serial order, weighted by the number of items within a series (e.g., two points per correct two-item series)
Towse et al., 2008	Read from computer; subjects provide a word to complete sentence	Completion word provided by subjects (integrated word condition), or unrelated target word provided (independent word condition)	None	Fail all three items in set	# of words correctly recalled

One way to directly confirm the involvement of working memory processes in speech-in-noise performance is to demonstrate training-related improvement in performance on speech-in-noise tests after increasing working memory capacity through training. A growing body of research has examined the efficacy of working memory training to improve working-memory capacity (for reviews, see Hindin and Zelinski, 2012; Melby-Lervåg and Hulme, 2013; Karr et al., 2014). The efficacy of working memory training, and cognitive training generally has been the subject of debate (e.g., Owen et al., 2010; Shipstead et al., 2012b; Jacoby and Ahissar, 2013; Lampit et al., 2014). However, if effective, working memory training (and cognitive training, in general), holds great promise for mitigating documented age-related declines in processing speed, episodic memory, working memory, and other domains of executive function (e.g., see Craik and Salthouse, 2007).

We examine whether working-memory training transfers to speech perception in noise in older adults, as well as to other tests of cognitive functioning. We used Cogmed Working Memory Training (Version QM: Pearson; Klingberg et al., 2002), an adaptive, computerized, commercial working memory-training program. We selected Cogmed since several publications have demonstrated its efficacy (Olesen et al., 2003; Klingberg et al., 2005; Holmes et al., 2009; Klingberg, 2010; Brehmer et al., 2011, 2012), and it includes a placebo training condition (an active control group). Improvements due to Cogmed training have been shown to generalize to related working-memory tasks (near transfer), including verbal working memory and visuo-spatial working memory, in both younger (Olesen et al., 2003; Klingberg et al., 2005; Holmes et al., 2009; Klingberg, 2010), and older (Brehmer et al., 2011, 2012) adults. Far transfer to other domains of intellectual functioning (verbal and non-verbal reasoning) has not been shown (Shipstead et al., 2012a). However, in a study of deaf children with cochlear implants, Kronenberger et al. (2011) observed improved verbal and non-verbal working memory capacity, as well as improved sentence-repetition ability after Cogmed training, providing some evidence of transfer to real-world speech comprehension ability, indicating suitability of the use of Cogmed for our study.

We tested for transfer to perception of speech in noise with two speech tasks. The first task was a closed-set, five-word sentence matrix test (BUG; Kidd et al., 2008), with two competing talkers (and one target speaker). The second task assessed perception in noise for sentences with and without supporting contextual information. The ability to use supporting contextual information to facilitate comprehension of degraded speech appears to depend on working memory (Janse and Jesse, 2014) and varies markedly among individuals (Rodd et al., 2005, 2012; Zekveld et al., 2012). It is not known, however, whether this benefit from context can be improved through working memory training.

**Figure 1:**
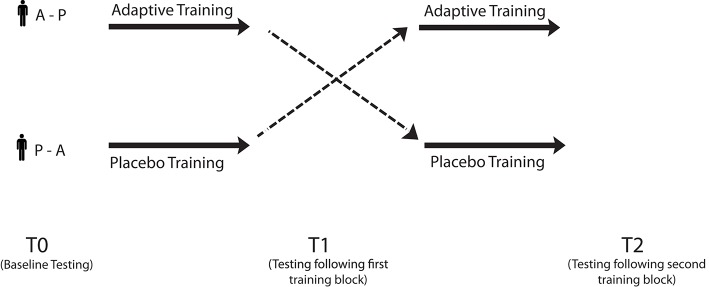
**Study design**. Participants were split into two groups and tested at three time points: at baseline, and following each of two 25-session training blocks. The A–P group received adaptive training followed by placebo training, whereas the P–A group received the placebo training followed by adaptive training.

**Figure 2:**
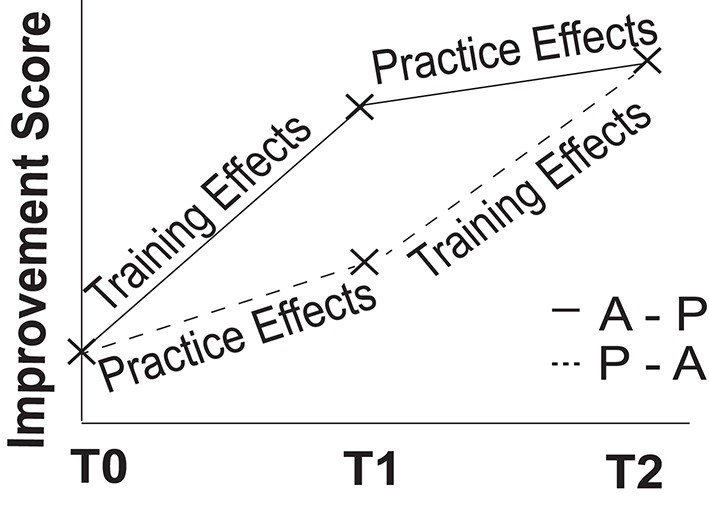
**Dissociating training from practice effects**. Our cross-over design allowed us to dissociate training effects from practice effects, within-subjects, by aggregating across the two 5-week blocks of training (T1-T0 and T2-T1). A–P refers to the group receiving adaptive training after baseline testing, followed by placebo training, whereas P–A refers to placebo training followed by adaptive training. Note that this is a hypothetical outcome.

We provided older adults with 5 weeks (25 sessions) of both adaptive and placebo Cogmed (Klingberg et al., 2002) training in a cross-over design, and evaluated cognitive functions at three time points: prior to the start of training (T0), following the first 5 weeks of either adaptive or placebo training (T1), and following the second 5 weeks of training (T2; participants who received adaptive training in T1 received placebo training in T2 and vice versa; see Figure 1). Cognitive evaluation included tests of working memory, processing speed, fluency, and short-term memory. We also assessed speech comprehension in noise using the BUG and high-/low-context sentence tasks. Near-transfer was assessed through improvements on other working memory tasks as a function of adaptive training, whereas far-transfer was operationalized as improvements on non-trained cognitive tasks (i.e., those other than tests of working memory, including speech tasks). Note that this design is among the first randomized active control trial for working memory training in older adults that specifically assesses for transfer to speech-in-noise function (see also, Henshaw and Ferguson, 2013 for the protocol for a forthcoming trial with hearing-aid users). Importantly, this design allows us to establish a causal link between working memory training and speech in noise function, rather than relying on correlational designs, a limitation of previous research. If working memory contributes to speech in noise perception, then improvements as a result of adaptive working memory training should lead to improved performance on tests of working memory (near-transfer), as well as on tests of speech perception, above and beyond practice effects (far-transfer; see Figure 2). In order to investigate the relationship between measures of working memory and other cognitive functions, and the ability to comprehend speech in noise, we examined correlations between cognitive abilities, particularly working memory ability, and speech-in-noise performance.

## Methods

### Subjects

We recruited 26 subjects (13 male, 13 female) between 59 and 73 years of age (mean = 64.96 years, *SD* = 3.77 years) through local newspapers, flyers, and community groups. Subjects generally reported good health and they were screened for hearing loss and mild cognitive impairment (see below) before beginning the study. Informed consent was obtained from all subjects and they were compensated for their time in the laboratory at a rate of $10 per hour. Subjects completing the study also received a $50 gift card for their efforts spent training online. The study was approved by the Queen's University Research Ethics Board.

### Screening procedure

Before commencing the study, all subjects received an audiogram (at 0.5, 1, 2, and 3 kHz), as well as the Montreal Cognitive Assessment (MoCA; Nasreddine et al., 2005). Subjects with scores of 23 or below on the MoCA were excluded from the study, as scores of 24 and above are indicative of cognitive impairment with over 95% sensitivity and specificity (Luis et al., 2009). We excluded two subjects (not reported here) on this basis. A total of 13 subjects were classified as having normal hearing, defined as thresholds at or below 25 dB HL at the tested frequencies. The 13 remaining subjects had some hearing loss: mild (a loss of < 40 dB in the better ear for at least one of the tested frequencies) in six participants; moderate (<55 dB in the better ear) in four; moderate-severe (<70 dB in the better ear) in two; and severe (>70 dB in the better ear) in one. These individuals were not excluded. The single participant with severe hearing loss wore hearing aids during testing, as did one participant with moderate hearing loss and one with moderate-severe hearing loss. We accounted for this heterogeneity in hearing levels by testing the effects of training in two ways: overall (collapsed across all subjects), as well as by conducting analyses for both groups separately (although this substantially reduces power).

During this initial screening session, subjects also completed portions of the Speech, Spatial, and Qualities (SSQ) of Hearing scale (Gatehouse and Noble, 2004; the subset of questions related to spatial hearing were not administered), Raven's Progressive Matrices (a measure of non-verbal intelligence), and a demographic questionnaire. Subjects also completed the Burns Anxiety Inventory (Burns, 1999); however, these scores did not correlate with other measures and were not used in subsequent analyses.

### Cognitive training procedure

Subjects were instructed to train 5 days a week for 10 weeks (i.e., 25 sessions in total for both active and placebo training) using the Cogmed working memory-training program (Klingberg et al., 2002). Twelve different training modules, involving remembering a sequence of numbers, letters, or objects for immediate recall, were used. Some exercises involved active manipulation of information, such as entering numbers in the reverse order that they appeared. Subjects worked on 8 of a possible 12 modules on each day of training; the modules that each subject had to complete on a given day were pre-determined by the online training program and were consistent across subjects. Training sessions took approximately half an hour to an hour per day to complete (with shorter times for placebo training).

In adaptive training modules, the level of difficulty was adjusted according to subjects' performance by increasing stimulus span length on the subsequent trial (conversely, span length was decreased following unsuccessful trials). In placebo training, only three items were ever presented for recall at a time. Since most individuals can easily recall three items, placebo training was not expected to improve working memory capacity (all subjects scored near 100% on placebo training). All subjects completed five blocked weeks of both adaptive and placebo training; however, the order in which the two different kinds of training (adaptive and placebo) was administered was pseudorandom and counterbalanced across subjects (see Figure 1). Two couples participated and in that case both partners were assigned to the same group at the same time, since we wanted to keep participants naive about the other condition they would perform. Subjects were told that they would be completing “brain training,” but no direct information about there being both adaptive and placebo conditions, and which condition they were completing at present, was provided.

The 25 sessions of each condition were completed on average in 33.88 days (*SD* = 2.86; including non-training days). The average time to completion did not differ significantly between the training groups or training type (placebo vs. adaptive) for subjects who successfully completed training. Progress was monitored remotely every week via the Cogmed Training Web to ensure training sessions were completed. Subjects received a weekly email from the experimenter (RVW; a certified Cogmed coach), who addressed training-related concerns and questions, and offered encouragement to maintain motivation.

Dependent variables from adaptive Cogmed training included the Start Index (calculated by Cogmed based on span length from training days 2 and 3), the Maximum Index (calculated by Cogmed from the two best days during training), as well as the Index-Improvement score (calculated by Cogmed as the Subtraction of Start Index from the Maximum Index).

### Testing procedure

The cognitive and speech tests were administered before and after each block of testing in two sessions, separated by a short break. The order of the sessions was identical for each subject across all three time-points, but counterbalanced across subjects. Cognitive testing was completed in a quiet room free of distractions. All auditory tests were conducted in a sound-attenuating booth (Eckel Industries) with headphones (Grado Prestige SR225). Speech stimuli were adjusted to a comfortable listening level based on feedback from subjects (mean = 76.49 dB, *SD* = 6.76). Speech levels for each subject were kept constant across all three time-points^1^. Testing was usually completed in 2.5–3.5 h (across two sessions), and at the same time of day, where possible.

### Cognitive test battery

We administered a broad cognitive test battery in order to assess the generalization of working memory training to multiple domains of cognitive function. Tests of near-transfer included measures of spatial working memory (Spatial Working Memory, CANTAB; Spatial Span Forward and Reverse, CANTAB), and verbal working memory (WAIS-IV Letter-Number Sequencing). Far-transfer tests included assessment of episodic memory (Paired Associate Learning Test, CANTAB), semantic fluency (Category Fluency; Strauss et al., 2006), response inhibition (Stop Signal Task, CANTAB), motor/processing speed (Reaction Time, CANTAB), and sustained visual attention (Rapid Visual Information Processing, CANTAB). We also included a computerized version of the Reading Span test (Rönnberg et al., 1989) and asked subjects to report the last word of every sentence. Tests were chosen on the basis of availability of published norms for older adults, although raw scores were used for the purposes of subsequent analyses. When multiple versions of a test existed (i.e., Paired Associate Learning, Stop Signal Task, Spatial Working Memory fluency), all three versions were counterbalanced across subjects. Please see Table 2 for further description of these tests and the dependent variables derived from them.

**Table 2:** **Summary of cognitive tests and outcome measures**.

**Cognitive Test**	**Domain Assessed**	**Description**	**Outcome Measures**
Spatial Working Memory (SWM)^*^	Working memory	Three, four, six, and eight boxes are dispersed on the screen. Subjects search for blue tokens hidden inside one of the boxes. Only one blue token is hidden at a time, without replacement (subjects must remember which boxes have produced a token).	Between Errors^**^—The number of times a box in which a token has previously been found is revisited. Strategy^**^—The number of times the subject begins a new search with a different box for six- and eight- box trials (note that this denotes an inefficient strategy).
Spatial Span (SSP) (forward and reverse modes)^*^	Working memory	White squares (boxes) are arranged in a variable sequence on screen. Subjects touch the boxes in the order in which they changed color. The length of the sequence begins at two and increases adaptively up to nine boxes. In reverse mode, subjects touch the boxes in the reverse order that they changed color.	The longest sequence successfully recalled by the subject, calculated for both the forward and reverse modes.
**WAIS-IV Letter-Number Sequencing**	Working memory	Subjects repeat back a string of letters and numbers in numerical order, followed by alphabetical order. The number of items in a string increases from 2 to 8 letters and digits.	*Total Score*—Number of items correctly reported, up to a maximum of 30. *Longest*—Longest string completed by a subject.
Reading Span	Working memory (complex test)	Subjects read aloud a series of unconnected sentences. After each sentence, subjects indicate whether the sentence made sense or not (e.g., “the girl sang a song” vs. “the train sang a song”) to prevent rehearsal of items. At the end of a series, they recall the last word of each sentence. The span of the series begins at 3 and increases to 6.	*Number of Correct Responses*—This is the sum of correct responses given for whether sentences were absurd or not. This score was used for validity purposes—a score of 85% correct or greater was deemed acceptable (which all subjects achieved). This score was not used in subsequent analyses. *Reading Span (Total)*—Total number of words correctly recalled. *Longest*—The longest series for which a subject was able to recall the last word of every item in the series.
Semantic/Category Fluency	Category fluency/processing speed	Subjects name as many animals, fruits, or vegetables as possible within 60 s.	Total number of correct items named.
Paired Associate Learning Test (PAL)^*^	Episodic memory	Subjects are presented with two, three, six, and eight boxes displayed on the screen that open one at a time in a randomized order to reveal a pattern. Respondents must select the box in which each pattern appeared.	Errors Adj.^**^—Total number of errors made, adjusting for each stage not attempted due to previous failure (the test discontinues if 10 consecutive errors are made at a stage). Errors, 8 Shapes, Adj.^*^—Total number of errors made at 8 shapes stage, adjusted if this stage is not reached.
Stop Signal Task (SST)^*^	Inhibition	Subjects make a two-choice button response, but withhold their response of a beep is heard on a trial. The timing of the auditory stop signal is set such that the subject is able to stop successfully approximately 50% of the time.	Direction Errors on Stop/Go Trials^**^—Number of trials in which the wrong button was pressed (left button when the right arrow was shown on screen and vice versa). *Proportion of Successful Stops (Last Half)*—The number of times the subject stopped successfully divided by the total number of stop signals during the last half of sub-blocks. Median Correct Reaction Time on Go Trials^**^—Median reaction time for Go trials (trials without a beep), in milliseconds. Stop Signal Delay (50%) (last half)^**^—Stop signal delay at which subject was able to stop 50% of the time. *Stop-Signal Reaction Time—*Time taken to respond.
Reaction Time (RTI)^*^	Motor/processing speed	Subjects respond to a yellow dot appearing on the screen. In simple reaction time, the dot appears in a circle in the center of the screen, and in five-choice reaction time, the spot appears in any one of five circles located concentrically to the center of the screen.	Five-choice Reaction Time^**^—Speed at which subject releases the press pad button in response to the appearance of the yellow dot during the five-choice reaction time task (speed of cognitive function). Five-choice Movement Time^**^—Time taken to touch the screen after the press pad button has been released during the five-choice reaction time task (speed of motor functions).
Rapid Visual Information Processing (RVP)^*^	Sustained visual attention	Digits from 2 to 9 appear in a box in the center of the screen in a pseudo-random order, at the rate of 100 digits per minute. Subjects are required to make a button press response to all of three target sequences (2-4-6, 3-5-7, or 4-6-8).	*A'—*A prime is the signal detection measure of sensitivity to the target, accounting for response bias.

This table lists cognitive tests repeated across the three time points, with a brief description of each test and outcome measures used. Bolded tests assess near-transfer (i.e., working memory ability), whereas the remainder of tests assess far-transfer to other cognitive domains. Tests with

* *are taken from the Cambridge Neuropsychological Test Automated battery (CANTAB)*.

Outcome measures with

** *are reverse coded (such that a lower value reflects a higher score)*.

### Speech tests

#### Sentence-matrix test (BUG)

Stimuli for this task were taken from Kidd et al. (2008). The words were recorded with neutral inflection so that all possible combinations of words could be used. Although the corpus consists of both male and female speakers, only eight female voices were used for this experiment. Every sentence had the structure <*name verb number adjective noun*>. Sentences consisted of a string of words composed by taking one word from each of the 5 categories (e.g., “Bob found three green shoes”). Stimuli were created by combining three distinct talkers (including two distractor voices), such that no word was repeated in any category across the three speakers. Subjects were instructed to follow the voice of the talker who said the name “Bob”; one signal with the name “Bob” was present in each trial. Items were mixed at 0 dB SNR. A MATLAB script was used to present the stimuli, at +3 and +6 signal-to-noise ratios (dB SNRs) in four blocks of 25 trials. The first block was provided as practice for subjects and was subsequently removed from analyses. Following each stimulus presentation, subjects were instructed to select each of the target words from a five by eight matrix of options (i.e., eight monosyllabic words from each of five different word-type categories), but the first column was not scored (since the target name was always “Bob”). Dependent variables were percentage of words correctly reported at both +3 and +6 dB SNR.

The closed set nature of the task ensures that contextual information and the load on working memory is constant across stimuli. This task has the advantage of having excellent psychometric properties (e.g., intelligibility is not confounded by a tendency to guess), and item effects are substantially weaker than for open-set materials, reducing within-subject variability, in principle making any change over time within subjects easier to detect.

#### Context sentences

We assessed the use of context by asking subjects to report words from sentences with and without contextual information, each presented with different sentences produced by two competing (same-sex) talkers. The task comprised of 96 semantically coherent (high-context) sentences, which had supportive contextual information (e.g., “He always read a book before going to bed”), and 96 semantically anomalous (low-context) sentences, which were syntactically correct but nonsensical and were created by replacing the content words of coherent sentences with other words matched in part of speech and word frequency (e.g., “Her good slope was done in carrot”; Davis et al., 2011). Distractor sentences consisted of common, everyday sentences (e.g., “the student tried to move the desk”).

Sentences were recorded by three individuals who were raised in southern Ontario and had an accent typical for the region (all were female; one individual recorded the target sentences and the other two recorded the distracter sentences). Sentences were divided into three sets of 64 sentences (32 at each level of context), matched across sets for average length. The order of the sentence sets was counterbalanced across participants, so that approximately equal numbers heard each set at each testing time point. Care was taken to avoid repetition of distractor sentences across sets where possible, but target sentences were never repeated (distractor sentences were never repeated within a set).

Stimulus mixing and presentation was accomplished using MATLAB. Target and distractor sentences were all normalized to have the same RMS power. The two distractor sentences for each trial were first combined and mixed at 0 dB SNR, then this result was normalized with the target and combined with the target at +3 and +6 dB. Subjects were instructed to attend to a target voice, identified as the voice to which they had listened during 10 practice sentences (no distractors were presented during practice). On each trial, subjects were instructed to type all of the words they could understand from the target sentence, in the correct order. Word report was assessed as the proportion of words correctly reported in each sentence. As in Wayne and Johnsrude (2012) and Davis et al. (2005), words were scored as correct if the written form perfectly matched the word produced in the sentence. Morphological variants were scored as incorrect, whereas homonyms and misspellings were scored as correct. Words were scored correct if they were reported in the correct order, even if intervening words were absent or incorrectly reported. All subjects correctly reported practice sentences (which included both high- and low-context sentences), indicating that word report is probably not limited by poor memory. Dependent variables were percentage of words correctly reported for both high- and low-context sentences at +3 and +6 dB SNR. Benefit from context was operationally defined as word report for high-context sentences minus word report for low-context sentences, at each SNR.

## Results

**Figure 3:**
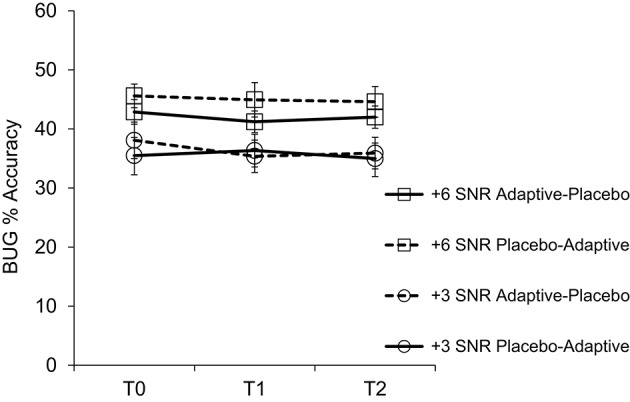
**Accuracy scores for the BUG speech task across the three study time points**. Error bars reflect standard error of the mean.

**Figure 4:**
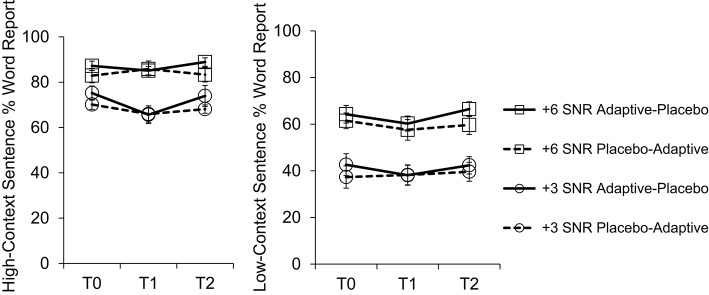
**Percentage word report scores for high- and low-context sentences across the three study time points**. Error bars reflect standard error of the mean.

Subjects in the two training groups (adaptive then placebo; and placebo then adaptive) did not significantly differ on any of the outcome measures reported in Table 3 at baseline (at T0). Two subjects (one couple) withdrew from the study for health reasons after completing 5 weeks of placebo training and the T1 test session; adaptive training data and T2 test data are unavailable for these subjects. Due to error, one subject completed 7 weeks of adaptive training followed by 3 weeks of placebo training. We included data from this subject, since more training may have increased the likelihood of finding any effect (which we did not observe anyway). Means and standard deviations for all outcome measures are reported in Table 3 (see Figures 3, 4 for accuracy and word-report data for speech tests). We first established the efficacy of Cogmed Working Memory Training by examining evidence of improvement on adaptive training. Training-related changes on the measures from the cognitive and speech-in-noise tests were then assessed using repeated-measures ANOVAs (Jacoby and Ahissar, 2013). Results are reported across all subjects, and for the normally hearing and hearing-impaired groups separately. Finally, we examine the correlations between speech tests and tests of working memory, and between working memory (including Reading Span) and other cognitive domains. Correlations were computed using Spearman's rho (unless otherwise noted) and all corrections were completed using the Benjamini-Hochberg Procedure.

**Table 3:** **Means and standard deviations for all outcome measures**.

	**Adaptive–Placebo**			**Placebo-Adaptive**		
	**T0**	**T1**	**T2**	**T0**	**T1**	**T2**
**SCREENING TESTS**						
Montreal Cognitive Assessment	27.85 (1.77)			27.15 (1.57)		
Raven (Raw Score)	50.92 (6.51)			51.27 (6.02)		
Speech Spatial Qualities: Speech	8.21 (0.77)			6.95 (1.24)		
Speech Spatial Qualities: Qualities	8.79 (0.55)			8.04 (0.89)		
**NEAR-TRANSFER (WORKING MEMORY) TESTS**						
Spatial Working Memory (Between Errors)	23.46 (15.59)	20.46 (13.35)	23.31 (13.79)	22.62 (10.97)	18.15 (13.67)	21.82 (11.30)
Spatial Working Memory (Strategy)	30.31 (7.47)	30.38 (5.94)	33.00 (5.34)	32.23 (4.34)	28.46 (5.17)	32.27 (4.33)
Spatial Span (Forward)	6.23 (1.17)	6.85 (1.46)	6.62 (1.12)	5.69 (0.95)	6.38 (1.33)	6.64 (1.21)
Spatial Span (Reverse)	5.62 (1.39)	6.54 (1.20)	6.69 (1.60)	5.85 (0.90)	5.70 (1.11)	6.36 (1.43)
Letter-Number Sequencing (Raw Score)	20.39 (2.14)	21.23 (2.49)	22.46 (2.93)	20.31 (1.60)	21.23 (2.49)	21.00 (1.18)
Letter-Number Sequencing (Longest)	6.00 (0.91)	6.15 (1.14)	6.62 (0.87)	5.61 (0.87)	5.85 (0.99)	5.82 (0.60)
**FAR-TRANSFER TESTS**						
Reading Span	21.15 (3.29)	24.65 (5.67)	26.69 (6.28)	21.84 (5.51)	24.85 (6.20)	23.10 (6.17)
Reading Span (Longest)	1.62 (1.56)	2.77 (1.30)	2.69 (1.25)	2.23 (1.59)	2.69 (1.25)	2.00 (1.61)
Fluency	19.31 (3.97)	22.00 (7.43)	20.62 (4.27)	16.54 (4.35)	18.08 (3.75)	22.73 (9.69)
Paired Associate Learning (Errors, Adj.)	18.08 (16.98)	14.92 (13.47)	11.77 (8.45)	16.54 (14.24)	16.31 (11.24)	16.18 (12.69)
Paired Associate Learning (Errors, 8 Shapes adj.)	13.92 (13.09)	11.15 (11.46)	9.08 (6.06)	12.00 (11.63)	12.15 (10.38)	10.64 (9.22)
Stop Signal Task (Direction Errors)	5.54 (12.35)	6.69 (10.03)	5.00 (4.62)	1.23 (1.96)	2.54 (4.13)	1.45 (1.86)
Stop Signal Task (Prop. Successful Stops)	0.51 (0.08)	0.51 (0.09)	0.52 (0.10)	0.56 (0.06)	0.54 (0.99)	0.55 (0.07)
Stop Signal Task (Median Correct, Go Trials)	540.77 (133.89)	503.85 (151.14)	489.92 (166.21)	568.04 (120.45)	548.88 (124.47)	537.41 (127.49)
Stop Signal Task (Stop Signal Delay)	323.73 (185.57)	284.24 (167.12)	292.06 (180.18)	351.58 (126.39)	365.86 (150.60)	372.84 (140.86)
Stop Signal Task (Reaction Time)	217.03 (78.00)	219.61 (58.69)	197.87 (44.61)	216.46 (55.57)	183.02 (44.78)	164.57 (33.75)
Reaction Time (Five-Choice Movement Time)	381.67 (96.93)	399.09 (89.58)	345.79 (99.88)	462.10 (123.27)	385.50 (98.41)	377.20 (89.79)
Reaction Time (Five-Choice Reaction Time)	336.98 (53.97)	334.08 (53.97)	311.97 (42.79)	350.85 (40.14)	342.64 (45.41)	309.78 (29.82)
Rapid Visual Processing (A′)	0.92 (0.05)	0.93 (0.03)	0.93 (0.04)	0.92 (0.06)	0.93 (0.05)	0.93 (0.06)
**SPEECH TESTS**						
BUG % Accuracy (+3 dB SNR)	38.07 (11.11)	35.36 (9.87)	35.90 (9.58)	35.47 (11.71)	36.35 (10.12)	34.97 (10.09)
BUG % Accuracy (+6 dB SNR)	42.87 (10.11)	41.20 (9.05)	41.99 (9.24)	45.59 (9.76)	44.93 (14.28)	44.59 (12.72)
Low-Context Sentences (+3 dB SNR)	42.62 (17.01)	38.09 (15.41)	42.39 (13.32)	37.33 (17.28)	38.26 (15.48)	39.61 (13.45)
Low-Context Sentences (+6 dB SNR)	64.25 (18.56)	60.25 (15.83)	66.46 (15.56)	61.46 (16.46)	57.56 (21.65)	59.68 (20.02)
High-Context Sentences (+3 dB SNR)	75.24 (11.26)	65.64 (13.97)	73.97 (16.71)	70.16 (18.20)	65.96 (23.65)	68.13 (23.34)
High-Context Sentences (+6 dB SNR)	87.23 (7.88)	85.11 (13.09)	88.90 (6.11)	82.92 (15.45)	85.82 (17.36)	83.33 (15.75)

*Note that reaction times are provided in milliseconds and speech scores are presented as percentage of words correctly reported (context sentences) or selected (BUG)*.

### Improvement on cognitive training (Cogmed)

The average Start Index (performance on days 2 and 3) was 85.38 (*SD* = 9.84, min = 70, max = 105) and the Maximum Index (performance on the two best training days) was 108.75 (*SD* = 13.53, min = 89, max = 142). The Cogmed Index-Improvement score, which compares these two (Maximum vs. Start), was 23.5 (*SD* = 7.95, min = 12, max = 49; the normal range is 18–42); this improvement was significant, and all subjects' scores improved over the course of adaptive training. The Index-Improvement score did not appear to depend on whether adaptive training was first or second (i.e., before or after placebo training). Crucially, the Index-Improvement score is comparable to (or larger than) those reported in studies reporting near-transfer (e.g., Gropper et al., 2014), suggesting that training was effective.

The average Raven's score was in the 67th percentile (minimum = 21st percentile, maximum = 99th percentile). The Raven's score significantly correlated with the Start Index score (*r*_*s*_ = 0.64, *p* = 0.001, corrected), but not with the Index-Improvement score or the Maximum Index, indicating that intelligence was related to initial performance on working-memory training, but not to training gains. The average Letter-Number Sequencing (a widely accepted test of verbal working memory) scaled score at the start of the experiment (T0) was 10.68 (*SD* = 1.92; note that 10 is the population mean scaled score, indicating average baseline working memory ability).

### Transfer to cognitive tests

For cognitive test outcome measures for which normative data were available (this excludes scores for Reading Span, Paired Associate Learning, 8-shapes Adjusted, Spatial Span Reverse, and the Stop Signal Task), the average *Z*-score across tests was 0.67 (*SD* = 0.19). On all tests, on average subjects performed within 1.5 standard deviations of the mean (min = −0.72, max = 1.37), indicating that older adults participating in our study consistently performed within acceptable limits.

We analyzed the data for each cognitive test using repeated-measures ANOVAs, with Training Group (adaptive before placebo or placebo before adaptive) as a between-subjects variable, and Time as a within-subjects variable with three levels. The Group by Time interaction reflects both training and the interaction between order (placebo, then training or training, then placebo) and training (either of which indicate that training has had some effect). The main effect of time reflects both practice, as well as training (see Figure 2). The main effect of training group was non-significant for all dependent variables, suggesting that participants in the two groups were drawn from the same population.

Significant practice effects (evident as a main effect of time with increasing values over time) were obtained on Reading Span, Letter-Number Sequencing score, Stop Signal Task Stop Signal Response Time, Spatial Working Memory Strategy score, Reaction Time Five-choice Movement Time and Five-choice Reaction Time, as well as Spatial Span Forward and Reverse (see Table 4 for statistics). The interaction between Group and Time was non-significant for all measures drawn from all the cognitive tests listed in Table 3, meaning that pre-post improvement did not differ between placebo and adaptive training, suggesting that the general improvements in performance are practice effects. Analyzing the hearing and hearing-impaired groups separately did not change the pattern of results. These results indicate no evidence for training-related cognitive improvement in older adults.

**Table 4:** *****F***-test statistics for main effect of time for all dependent variables, and (uncorrected) ***post-hoc*** comparisons**.

**Dependent Variable**	***F*_(2, 44)_**	***p***	**η*_*p*_*^2^**	*Post-hoc* comparisons ^*^p < 0.05; ^**^p < 0.001
Reading Span Score	13.26	<0.001	0.38	T2 > T0^**^, T1 > T0^**^
Letter-Number Sequencing Score	5.81	<0.05	0.21	T2 > T0^*^
Stop-Signal Task Stop Signal Reaction Time	4.46	<0.05	0.17	T2 > T0^*^
Spatial Working Memory Strategy Score	3.16	0.052	0.13	T2 > T1^*^
Reaction Time Five-Choice Movement Time	6.19	<0.05	0.22	T2 > T0^*^
Reaction Time Five-Choice Reaction Time	11.23	<0.001	0.34	T2 > T0^**^, T2 > T1^**^
Spatial Span Forward Score	3.94	<0.05	0.15	T2 > T0^*^
Spatial Span Reverse Score	4.37	<0.05	0.17	T2 > T0^*^
High Context Sentences +3 dB SNR	8.61	<0.001	0.28	T0 > T1^*^

We conducted additional exploratory analyses on training-related transfer, using the baseline score and training group (adaptive vs. placebo) as separate predictors in a linear regression. Data were recoded to match a between-subjects design, such that each subject contributed two data points for each of post-training performance and baseline performance, with a dummy variable coding for adaptive vs. placebo training (e.g., For the adaptive-placebo group, T0 was taken as baseline in the adaptive condition, and T1 was baseline for the placebo condition, with T1 as the post-training performance in the adaptive condition, and T2 for the placebo condition). Although this analysis was more sensitive than the repeated-measures ANOVA, we did not find any significant effect of training group, even at an uncorrected level.

### Transfer to speech tests

Data for speech tests are presented in Figures 3, 4. As expected, the effect of SNR was significant for all three tasks, with +6 dB SNR being more intelligible than +3 dB SNR (see Table 3). In addition, significantly more words were reported for high-context sentences (proportion of words reported correctly: *M* = 0.78, *SD* = 0.14, min = 0.34, max = 0.93) than for low-context sentences (*M* = 0.52, *SD* = 0.15, min = 0.17, max = 0.74; *t*_(25)_ = 16.81, *p* < 0.001; similar to (Davis et al., 2011)). The average proportion of words correctly reported on the BUG at +6 dB SNR was 0.44 (*SD* = 0.10, min = 0.28, max = 0.70) and 0.36 (*SD* = 0.09, min = 0.22, max = 0.56) at +3 dB SNR. These results suggest that performance was not at ceiling or floor for either of the speech tasks. We conducted a repeated-measures ANOVA on BUG performance and on word-report scores for high- and low-context sentences separately, as well as on the context benefit (high-low) score, with training group as a between-subjects factor and time and SNR (+3 dB and +6 dB) as within-subjects factors. Speech scores did not improve as a result of training (far-transfer); the interaction between Group and Time was non-significant for all three tasks. We also analyzed high- and low-context scores together; even with increased power, there was no effect of training group. A lack of training-related transfer to speech tests is unsurprising given the lack of transfer to other cognitive tests. The main effect of Group was non-significant across all three tasks, but there was a main effect of Time for high-context sentences presented at +3 dB SNR [*F*_(2, 44)_ = 8.61, *p* < 0.001, η${}_{p}^{2}$= 0.28], reflecting practice effects. Conducting analyses separately on hearing and hearing-impaired subjects did not change the overall pattern of results, and, similar to cognitive tests, our exploratory analyses (see previous section for details) did not reveal any significant training-related effects.

### Correlations between speech and cognitive tests

Since scores on cognitive tests did not depend on training (as demonstrated in a previous section), participants' scores on each test were averaged across all three time points (and across the two SNRs for the BUG and high- and low-context sentence tests) to yield more reliable estimates of performance.

We computed correlations between average context-benefit scores (difference in word report for high- and low-context sentences) and tests of working memory (see Table 5). None of the correlations with context benefit survived correction. Although there was a trend toward significance for Reading Span Longest score (*r*_*s*_ = −0.42), Letter-Number Sequencing score (*r*_*s*_ = −0.41), and Letter-Number Sequencing Longest score (*r*_*s*_ = −0.44), the negative direction of these trends was contrary to expectations, as they appeared to be driven by a positive relationship between working memory and intelligibility of low-context sentences specifically. In fact, word report for low-context sentences significantly correlated with Letter-Number Sequencing Average score (*r*_*s*_ = 0.58, *p* < 0.05) and Letter-Number Sequencing Longest score (*r*_*s*_ = 0.48, *p* < 0.05, both corrected). There was also a trend toward significance for correlations between low-context sentence word report and Reading Span total (*r*_*s*_ = 0.45) and Longest scores (*r*_*s*_ = 0.39; these did not survive correction for multiple comparisons). There was no relationship between word-report for high-context sentences and tests of working memory, even when examined at only the more difficult +3 dB SNR to minimize ceiling effects, with the exception of a trend in the predicted direction for Spatial Working Memory Errors (*r*_*s*_ = −0.38; the apparent negative correlation reflects the fact that low values are indicative of better performance). The difference between correlations for low- and high-context sentences was significant for both Letter-Number Sequencing Average score (Steiger's Z-test; *Z* = 2.48, *p* < 0.05) and Letter-Number Sequencing Longest score (*Z* = 2.70, *p* < 0.05).

**Table 5:** **Correlations between speech tests and (select) cognitive tests**.

	**RS**	**RS**	**LNS**	**LNS**	**SWM**	**SSP**	**SSP**	**PAL**	**SST**	**Raven's**	**BUG**	**H**	**L**	**H-L**
		**L**		**L**	**Errors**	**Fwd**	**Rev**	**(8)**	**SS**			**Context**	**Context**	**Context**
**RS**														
**RS L**	0.81^**^													
**LNS**	0.43^*^	0.37												
**LNS L**	0.36	0.33	0.90^**^											
**SWM (Errors)**	−0.16	−0.12	−0.44^*^	0.23										
**SSP Fwd**	0.31	0.28	0.46^*^	0.46^*^	−0.33									
**SSP Rev**	0.37	0.26	0.61^**^	0.51^**^	−0.30	0.54^**^								
**PAL (8)**	−0.54^**^	−0.36	−0.66^**^	−0.69^**^	0.32	−0.32	−0.31							
**SST SS**	−0.34	−0.27	−0.21	−0.37	0.17	−0.02	−0.03	0.28						
**Raven's**	0.27	0.14	0.46^*^	0.29	−0.45^*^	0.23	0.61^**^	−0.28	−0.07					
**BUG**	0.16	−0.01	0.18	0.09	−0.12	−0.07	0.16	−0.12	−0.01	0.24				
**H Context**	0.31	0.14	0.26	0.11	−0.34	−0.23	0.06	−0.17	−0.06	0.33	0.55^**^			
**L Context**	0.45^*^	0.39	0.58^**^	0.48^*^	−0.24	0.04	0.22	−0.42^*^	−0.10	0.27	0.47^*^	0.76^**^		
**H-L Context**	−0.20	−0.42^*^	−0.41^*^	−0.44^*^	−0.13	−0.19	−0.13	0.25	−0.06	0.04	0.11	−0.00	−0.50	

*Note that significance levels displayed here are uncorrected for multiple comparisons. RS (Reading Span), RS L (Reading Span Longest Span), LNS (Letter-Number Sequencing), LNS L (Letter-Number Sequencing, Longest Sequence) SWM (Spatial Working Memory), SSP Fwd (Spatial Span Forward), SSP Rev (Spatial Span Reverse), SST SS (Stop Signal Task, Proportion of Successful Stops), BUG (Sentence-matrix speech in noise task), H Context (High-Context sentence word report scores), L Context (Low-Context sentence word report scores), H-L Context (High – low context sentence word report scores)*.

* significant at p < 0.05;

** *significant at p < 0.01*.

This pattern of results suggests that working memory may facilitate intelligibility particularly when contextual information is unavailable, although this may be an artifact of the word-report intelligibility measure we used. Word report is a rather unnatural assessment of speech intelligibility. Listeners generally do not need to repeat back sentences in everyday communication, and it is possible that word report scores for low-context sentences may load more highly on working memory because they are harder to remember than high-context sentences. We evaluated this hypothesis by examining the Pearson correlation between sentence length and word-report scores, for high- and low-context sentences separately. These were then averaged, within-subjects, across test time points. A repeated-measures ANOVA, with high- vs. low-context sentences and time as within-subjects factors, and training group as a between-subjects factor revealed a main effect of level of context [*F*_(1, 22)_ = 30.17, *p* < 0.001, η^2^ = 0.58]: sentence length was significantly more negatively correlated with word report for low- compared to high- context sentences (low-context *M*_*r*_ = −0.26, *SE* = 0.02; high-context *M*_*r*_ = −0.08, *SE* = 0.03). The interaction between Context Level and Time trended toward significance (*p* = 0.05, sentence word report for low-context sentences was significantly more negatively correlated with sentence length at all three time points). All other main effects and interactions were non-significant. This result is consistent with low context sentences being more difficult to maintain in memory, and may account for the correlation between low-context sentences and working memory.

We did not observe any significant correlations between working memory measures and the BUG speech task scores. Interestingly, scores on the speech tasks did not significantly correlate with the Raven's nor with the SSQ Speech or SSQ Qualities measures, although there was a trend for a correlation between SSQ Speech and low-context sentence word-report scores (*r*_*s*_ = 0.36).

### Correlations between reading span and cognitive measures

Correlations between Reading Span score (i.e., the total number of words recalled) and speech-in-noise performance in previous reports have generally been taken as evidence for the involvement of working memory in speech-in-noise performance (Tun et al., 1991; Akeroyd, 2008; Zekveld et al., 2012; Besser et al., 2013; Zekveld et al., 2013; Davies-Venn and Souza, 2014, but see Humes and Coughlin, 2009; Schoof and Rosen, 2014). However, the Reading Span test is a complex test that relies on other cognitive domains, in addition to working memory. We examined the cognitive architecture supporting Reading Span performance by correlating measures on this test with other measures of working memory (Letter-Number Sequencing Average score, Spatial Working Memory score, Spatial Span Forward, and Spatial Span Reverse scores), non-verbal reasoning (Raven's), processing speed (Reaction Time Five-choice Reaction Time), memory (Paired Associate Learning, 8-Shapes Corrected score) and inhibition (Stop Signal Task Proportion of Successful Stops). As seen in Table 5, the Reading Span score correlated with the Paired-Associate Learning, 8-Shapes Total Errors Adjusted score (*r*_*s*_ = −0.54, *p* < 0.05, corrected for multiple comparisons). There was a trend toward significance for the correlations between Reading Span score and Letter-Number Sequencing score (*r*_*s*_ = 0.43), Spatial Span Reverse (*r*_*s*_ = 0.37), as well as Stop Signal Task Proportion of Successful Stops (*r*_*s*_ = −0.34, *p*>0.05, after correction for multiple comparisons). The lack of significance here is probably due to insufficient power, but the pattern of results indicates, not surprisingly, that the Reading Span Test loaded on tests of working memory, episodic visual memory, and inhibition.

## Discussion

### Generalizability of cognitive training

As expected, scores on the Cogmed working-memory tests increased over time in the adaptive-training sessions, and both the adaptive and placebo training groups showed practice effects on several test measures. However, we observed no evidence of transfer of working-memory training, even to tests that should tap the same cognitive domains as training (i.e., other working memory tests), and even when uncorrected for multiple comparisons. Ultimately, our results demonstrate no evidence that Cogmed cognitive training improved cognitive functioning as measured by our tests, or improved speech-in-noise comprehension in older adults.

Our results apparently contradict those documenting near- or far-transfer of working memory training, including Cogmed, to other cognitive domains in both older and younger adults (Olesen et al., 2003; Klingberg et al., 2005; Holmes et al., 2009; Klingberg, 2010; Brehmer et al., 2011, 2012; Kronenberger et al., 2011; Hindin and Zelinski, 2012; Melby-Lervåg and Hulme, 2013; Karbach and Verhaeghen, 2014; Karr et al., 2014). In attempting to reconcile our null results for generalization of cognitive training with significant generalization noted elsewhere, it is important to note that effects of training may be specific to assessment measures used. Others have suggested that even in the absence of direct strategy instruction, cognitive strategies may be task specific (Dunning and Holmes, 2014), reflecting limited generalizability of training gains. Alternatively, Cogmed training may specifically benefit those with below-average working memory ability (Zinke et al., 2014). The vast majority of our subjects had at least average working memory ability (on Letter-Number Sequencing, all subjects had baseline scores at or above the 25th percentile (the low-average range), and our subjects performed on average, half a standard deviation better than the norm on cognitive tests).

Although a lack of motivation could in principle explain a lack of efficacy, our older participants exhibited acceptable improvement scores on training, commensurate with studies reporting evidence of transfer (Gropper et al., 2014) and they appeared highly motivated. We did not compensate them for training time; only for the time spent in the testing sessions, and we experienced a very low rate of attrition. It is also possible that our study was underpowered. However, our study had more than the mean number of adults assessed in the studies reviewed in the meta-analysis of cognitive training studies in older adults by Karbach and Verhaeghen (2014; 21.34; *SD* = 13.98); this analysis revealed significant effects for training, although none of the studies used Cogmed (these studies also typically used a between-subjects design, a less powerful design than our within-subjects design).

Instead, our results are consistent with an emerging body of literature challenging the effectiveness and generalizability of cognitive training, including Cogmed (e.g., Shipstead et al., 2012a,b; Chacko et al., 2013; Jacoby and Ahissar, 2013; Melby-Lervåg and Hulme, 2013; Gathercole, 2014; Lampit et al., 2014). At present, the reasons for inconsistencies between these studies and those reporting evidence of generalization (e.g., Kronenberger et al., 2011) is unclear. However, a recent meta-analysis and review of cognitive training in 5000 older adults found that home-based training was ineffective compared to group-based training, and that training more than three sessions a week was less effective than training three or fewer times per week, perhaps due to cognitive fatigue (Lampit et al., 2014). Our subjects trained five times per week at home, which may account for our null findings. However, it is also important to note that effect sizes reported in this meta-analysis are small (Hedge's *g* = 0.22).

Our cross-over, within-subjects design appears to be unique in the literature; to our knowledge our design in which subjects receive *both* placebo and adaptive training (in a counterbalanced manner) has not been used in studies evaluating the efficacy of Cogmed Working Memory Training (Olesen et al., 2003; Klingberg et al., 2005; Holmes et al., 2009; Klingberg, 2010; Brehmer et al., 2011, 2012; Kronenberger et al., 2011; Hindin and Zelinski, 2012; Chacko et al., 2013; Melby-Lervåg and Hulme, 2013; Karr et al., 2014), or any other form of cognitive training in older adults. This design is more rigorous than the traditional between-subjects approach because it controls for motivational/engagement effects resulting from training. More specifically, it is possible that motivation and engagement is higher in the training group, owing to the higher degree of effort necessitated by the training regimen, compared to even an active placebo group (since the task performed by the placebo group is usually easier). Thus, where adaptive and placebo training are not counterbalanced within subjects, the adaptive group may show training gains simply as a result of effort and engagement, rather than cognitive training (see also Jacoby and Ahissar, 2013 for a similar argument).

This view is supported by the apparent absence of significant differences between active (placebo training) and passive (no training) control groups in other studies (see Melby-Lervåg and Hulme, 2013; Karbach and Verhaeghen, 2014 for reviews). This finding that placebo training is equivalent to no training at all suggests that the placebo condition in Cogmed is not sufficiently demanding to control for effects related to effort or engagement during training. Thus, it is possible that evidence of transfer of training to cognitive tests in studies comparing adaptive to placebo (or passive training) might reflect gains due to task engagement, rather than the content of the training regimen, suggesting that these gains might be acquired through engagement with non-specific cognitive tasks. Moreover, the relative superiority of group-based (or lab-based) training over at-home training (Lampit et al., 2014) also suggests that task-engagement, or social interaction, may drive cognitive training gains.

### Relationship between working memory and speech in noise

We observed that working memory measures correlated with the amount of benefit to word-report obtained through provision of greater context, but this relationship is explained by a positive correlation specifically with low-context word-report scores. The cognitive load imposed by low-context (semantically anomalous) sentences (as listeners strive fruitlessly after a coherent meaning) may limit processing resources available for accurate perception of further words in the utterance, reducing word report. The greater the cognitive capacity, the more resources are left over from this futile semantic integration process for accurate perception. Moreover, low-context sentences may place a greater strain on working memory since the lack of meaningful associations among the words in the sentences means that fewer retrieval cues for any given word are available. As word report was higher for high-context sentences compared to low-context sentences, it is also possible that low-context sentences were more effortful (as reflected by greater recruitment of working memory resources) as a result of being more difficult to maintain in memory for immediate report. This explanation is supported by our finding of a significant negative association between sentence length and word report for low-context sentences but not for high-context sentences. It is possible that correlations between working memory and word report for high-context sentences may emerge more strongly in longer streams of perceptual inputs (i.e., longer utterances) due to increased processing demands.

### What does the reading span test measure?

Our results warrant some caution in using the commonly administered Reading Span test (Rönnberg et al., 1989) as an exclusive test of working memory. The Reading Span test correlated with memory, with a trend for correlation with measures of working memory and inhibition. The pattern of results suggests that Reading Span may load on both working memory and general cognitive functioning, including episodic visual memory. Given the documented contributions of more general cognitive functioning to speech perception (e.g., Wingfield and Tun, 2007; Arlinger et al., 2009; Heald and Nusbaum, 2014), as well as the high correlations between working memory and non-verbal intelligence (e.g., Engle et al., 1999), future research should take care to parcel contributions of working memory from other cognitive processes. This can be achieved by using more domain-specific tests of working memory (e.g., WAIS Letter-Number Sequencing and other simple span tests), as well as using multiple, converging measurements of working memory. It would also be worthwhile to compare the widely-used Rönnberg et al. (1989) version of the Reading Span test with the Daneman and Carpenter (1980) version (as well as others) to verify whether they can justifiably be used interchangeably.

## Summary and future directions

Commercial cognitive training software, including Cogmed Working Memory Training (Klingberg et al., 2002), is being aggressively marketed to the general population. Our study adds to the growing body of literature suggesting that cognitive training, a multi-million dollar industry, may not be as effective as initially hoped (e.g., Shipstead et al., 2012a; Melby-Lervåg and Hulme, 2013). Our study lends further support to the idea that working memory is important in speech comprehension. Although, our results suggest that individuals with better working memory capacity may be better able to compensate for degraded auditory input when contextual information is unavailable, this may be an artifact of our word report measure. Future studies should extend these findings to more naturalistic paradigms, such as through comparing reaction time as a function of cognitive load in dual-task paradigms for high- and low-context sentences. Our study also suggests that exclusive reliance on the Reading Span as a measure of working memory may be problematic, since in our study Reading Span correlated significantly only with a measure of episodic visual memory, but not working memory. Future research should also aim to evaluate the impact of cognitive training on everyday cognitive functioning, also controlling for levels of effort, which are typically not matched in an active, low-level task control group (including the placebo condition of Cogmed). Working memory training, if effective, would be a cornerstone of rehabilitation programs for older adults with communication difficulties, but the evidence suggests that we have not yet found the magic ingredient.

## Author contributions

JJH, IJ, and RW designed the study. Stimulus creation, data collection, and data analysis were conducted by CH, JJ, and RW. RW wrote the manuscript; IJ edited the manuscript and CH and JJH commented on the final version.

## Funding

This research was funded through an NSERC Discovery Grant and CIHR Operating Grant to IJ.

### Conflict of interest statement

The authors declare that the research was conducted in the absence of any commercial or financial relationships that could be construed as a potential conflict of interest. The reviewer AB and handling Editor declared their shared affiliation, and the handling Editor states that the process nevertheless met the standards of a fair and objective review.

## Footnotes

^1^For eight participants, the levels were changed due to experimental error at T1 and T2. However, at T2, the average change in levels was identical between the A–P and P–A groups for both speech tasks. At T1, the change in levels favored the adaptive group; this biased the results in favor of improved speech comprehension performance as a result of cognitive training, which we still did not observe.
